# Cough-induced chylothorax in a two-year-old boy – case report and review of the literature

**DOI:** 10.1186/s12887-023-04221-9

**Published:** 2023-08-23

**Authors:** Melanie Anger, Julian Hofmann, Bettina Ruf, Marc Steinborn, Daniela Reber, Katharina Warncke, Nikolaus Rieber

**Affiliations:** 1 Department of Pediatrics, Kinderklinik München Schwabing, Munich Klinik and School of Medicine, Technical University of Munich, Kölner Platz 1, 80804 Munich, Germany; 2 Department of Pediatric Cardiology and Congenital Heart Disease, German Heart Center Munich, Technical University of Munich, Munich, Germany; 3 Department of Diagnostic and Interventional and Pediatric Radiology, Kinderklinik München Schwabing, Munich, Germany

**Keywords:** Chylothorax, Pleural effusion, Octreotide, Children, Traumatic

## Abstract

**Background:**

Chylothorax is a very rare form of pleural effusion in children, especially after the neonatal period, and predominantly occurs secondary to cardiothoracic surgery. It can lead to significant respiratory distress, immunodeficiency, and malnutrition. Effective treatment strategies are therefore required to reduce morbidity.

**Case presentation:**

A previously healthy two-year old boy was admitted with history of heavy coughing followed by progressive dyspnea. The chest X-ray showed an extensive opacification of the right lung. Ultrasound studies revealed a large pleural effusion of the right hemithorax. Pleural fluid analysis delivered the unusual diagnosis of chylothorax, most likely induced by preceded excessive coughing. After an unsuccessful treatment attempt with a fat-free diet and continuous pleural drainage for two weeks, therapy with octreotide was initiated. This led to complete and permanent resolution of his pleural effusion within 15 days, without any side effects.

**Conclusions:**

Severe cough may be a rare cause of chylothorax in young children. Octreotide seems to be an effective and safe treatment of spontaneous or traumatic chylothorax in children.

There is, however, a lack of comprehensive studies for chylothorax in children and many issues concerning diagnostic strategies and treatment algorithms remain.

## Background

Chylothorax in children beyond the neonatal period is a very rare condition with an estimated prevalence of 1:15,000 Children [1, 2].

Complications include respiratory failure, malnutrition, Hypovitaminosis and immunodeficiency which can lead to serious illness and death if not adequately treated [3, 4]

**Fig. 1:**
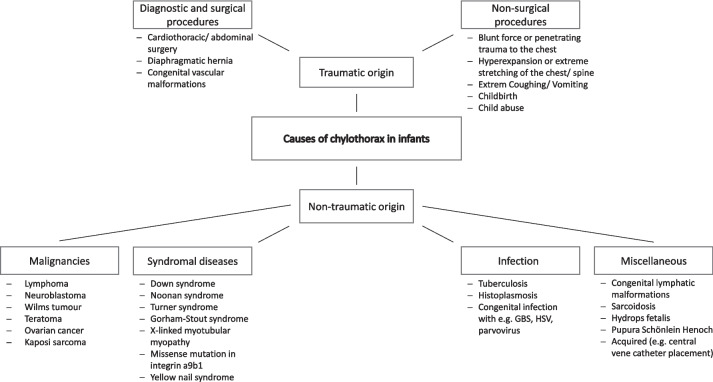
Overview of the most common causes of chylothorax in children [1, 26, 36–38]

A chylothorax is defined as the accumulation of chyle in the pleural space due to thoracic duct leak [5]. The etiology can be either traumatic or non-traumatic, traumatic causes comprise up to 54% and are secondary to either surgical (the majority of cases) or non-surgical events (ca. 3%) [6, 7]. In regards to non-surgical traumatic events, a few reports suggest injury to the thoracic duct due to elevated intrathoracic pressure after severe episodes of vomiting, coughing or delivery maneuvers [8, 9]. Non-traumatic causes include malignancies, most commonly lymphoma [5, 7], infectious diseases such as tuberculosis or others including subclavian vein thrombosis and lymphatic anomalies (Fig. 1) [10–12]. In pediatric patients most episodes of chylothorax are secondary to cardiothoracic surgery [11, 13], followed by nonsurgical traumatic events and malignancy [10].

Chyle is an alkaline, bacteriostatic milky fluid, which primarily consists of triglycerides and proteins as well as lymphocytes, with an electrolyte content similar to plasma (Table 1) [14].

**Table 1:** Chyle: composition and characteristics. [1, 26, 36]

Character	Milky, opalescent, serous
Culture	Sterile
pH	> 7,2
Glucose	> 60 mg/dl
Total fat	0,4 – 6 g/dl
Cholesterol	65 – 220 mg/dl
Triglycerides	> 110 mg/dl
Chylomicrons	Present
Total protein	2 – 6 g/dl
Albumin	1,2 – 4,2 g/dl
Globulin	1,1 – 3,1 g/dl
Cell count	> 1000 cells/l
Erythrocytes	50 – 600/mm^3^
Polymorphonuclear Leukocytes	~300/mm^3^
Lymphocytes	400 – 6800/mm^3^
Electrolytes	
Sodium	104 – 108 mmol/l
Potassium	3,8 – 5 mmol/l
Chloride	85 – 130 mmol/l
Calcium	3,4 – 6,0 mmol/l
Phosphate	0,8 – 4,2 mmol/l

With ongoing chyle loss, large volumes of fluid, immunoglobulins, lymphocytes, and coagulation factors (specifically factor VII and fibrinogen), get lost and may cause immunosuppression, coagulation disorders, electrolyte imbalance, and metabolic acidosis [15]. Effective treatment strategies are therefore urgently required. Here, we describe and discuss the clinical presentation, diagnosis, and treatment of chylothorax presumably caused by heavy coughing in a young boy.

### Case presentation

A two-year-old boy was referred to our emergency department by his pediatrician due to progressive dyspnea, asymmetric breathing sounds by auscultation and fatigue. The boy was born at term and had no pertinent past medical history.

During the 15 days prior to hospitalization, the boy suffered from a heavy paroxysmal cough without fever. Outpatient treatment consisted of salbutamol inhalation and systemic prednisolone administration leading only to short-term alleviation of his symptoms.

At the time of admission, clinical examination revealed symptoms of respiratory distress with intercostal and subcostal retractions. Breathing sounds over the right lung were absent. Vital signs were stable and venous blood gas analysis did not show signs of respiratory insufficiency.

**Fig. 2:**
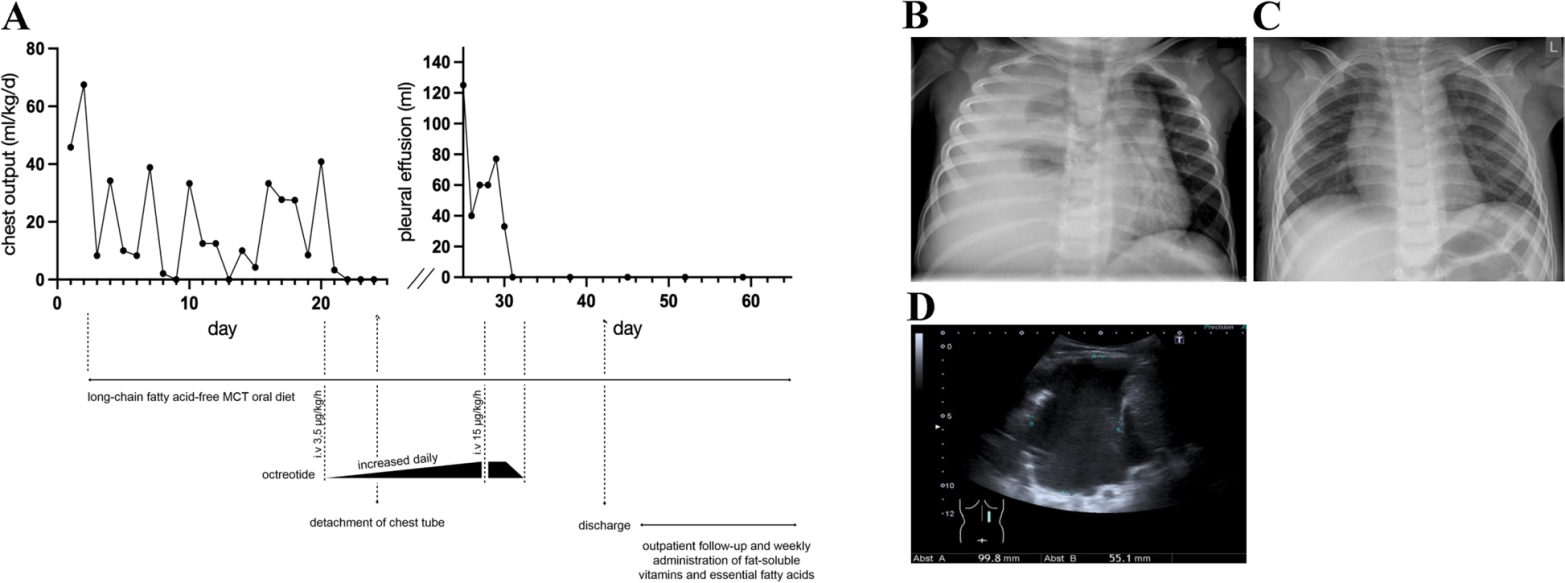
Overview on clinical course and radiology in our patient. **A** Chest-tube output (ml/kg/d) before chest-tube detachment and estimated pleural effusion volume via ultrasound (ml) after chest-tube detachment in relation to therapy. Long-chain fatty acid free MCT diet was initiated after confirmation of chylothorax at day two. Parenteral octreotide therapy was started at day 20 due to a remaining chest output of 70 ml/kg/d. Daily drainage output volume decreased under therapy without relapse after tapering octreotide at day 32 and stopping oral diet at day 64. **B** Chest X-ray on admission showing extensive opacification of the right hemithorax and mediastinal shift to the left. **C** Chest X-ray after termination of chest-tube drainage with almost complete resolution of pleural effusion. **D** Ultrasound demonstrates right-sided pleural effusion with internal echos and caudal shift of the diaphragm and liver

Chest X-ray showed a complete white-out of the right hemithorax with left mediastinal shift (Fig. 2B). Ultrasound studies revealed an extensive right-sided pleural effusion with caudal shift of the diaphragm and liver (Fig. 2D). The boy was admitted to our intensive care unit for close monitoring and further evaluation. A contrast enhanced CT Thorax displayed no evidence of malignancy, no signs of pleural inflammation, thrombosis, contrast medium leak or lymphatic malformation. We performed a thoracentesis with fractioned pleural fluid aspiration of 1200 ml milky-cloudy fluid.

The biochemical analysis of the pleural fluid revealed high levels of triglycerides (900 mg/dl, serum level at 83 mg/dl), total protein (4,8 g/dl), and lactate dehydrogenase (610 U/l, serum level at 494 U/l). The ratio of pleural fluid cholesterol to serum cholesterol was < 1.0, further supporting the suspected diagnosis of chylothorax. Cytology showed an absolute cell count of 6820 cells/mm^3^, almost exclusively lymphocytes (99%) and no signs of malignancy. Based on these findings we established the diagnosis of a chylothorax. In line with the suspected diagnosis blood tests revealed low levels of gamma globulins and Antithrombin 3 as well as hypalbuminemia.

Initial treatment included continuous suction-free tube thoracostomy and a fat-free diet with supplementation of medium-chain triglycerides. As the leakage did not resolve under this protocol within 20 days (max. chest drainoutput 70 ml/kg/day), parenteral therapy with octreotide was initiated. The drug was given as continuous intravenous infusion with a starting dose of 3,5 µg/kg per hour, which was increased daily over six days to a maximum dose of 15 µg/kg per hour. Under this regime, the drainage volume reduced gradually (Fig. 2A and D) and we tapered the octreotide therapy over a further three days. Of note, on day four of octreotide therapy the chest tube became accidentally detached. Since we had already observed a good therapeutic response, we decided not to insert another drain and the residual pleural fluid was monitored closely via ultrasound. The remaining 125 ml of chyle completely resolved after eleven days of octreotide therapy.

Since adverse effects of octreotide therapy may include hypo- or hyperglycemia, thyroid dysfunction, muscular cramps, nausea, renal impairment, and liver dysfunction [16], we initially monitored blood sugar levels every three hours and determined electrolytes, thyroid hormones, transaminases, albumin, fat-soluble vitamins, immunoglobulin levels and coagulation parameters weekly. No adverse effects occurred and the hypogammaglobulinemia, hypalbuminemia and loss of Antithrombin 3 completely resolved at day seven of octreotide therapy.

Parallel to octreotide therapy, an accompanying weekly intravenous substitution of fat-soluble vitamins and essential fatty acids was initiated during the admission and was continued in our outpatient day-clinic for a total of five weeks. The boy did not exhibit any weight loss or malnutrition and remained in a good general health condition throughout the course of treatment. Within nine months, no relapse occurred.

## Discussion and Conclusions

**Fig. 3:**
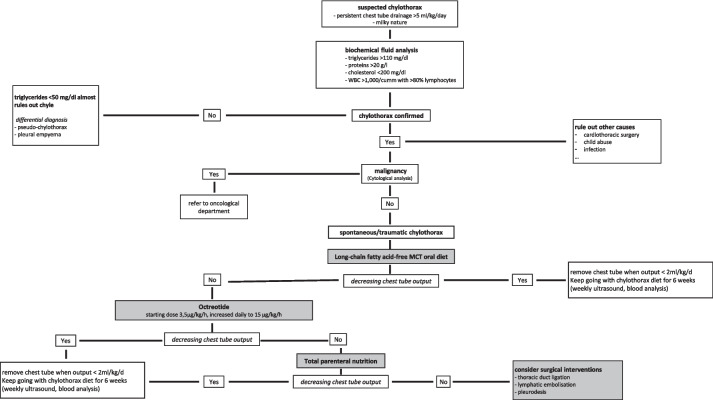
Our algorithm for diagnosis and treatment of chylothorax in young children

This case report presents excessive coughing as a probable cause of chylothorax and suggests a successful multimodal treatment strategy. There is a lack of comprehensive studies for chylothorax in children and many issues concerning diagnostic strategies and treatment algorithms remain (Fig. 3).

For the proper diagnosis of chylothorax biochemical fluid analysis is essential. Pseudo-chylothorax, which has similar appearance and consistency, needs to be distinguished due to the completely different therapeutic approaches required. The latter is commonly associated with chronic inflammatory diseases such as rheumatoid arthritis and is cholesterol-rich (cholesterol > 200 mg/dL, triglyceride < 110 mg/dl, cholesterol/triglyceride ratio > 1 and pleural/serum cholesterol ratio > 1) when compared to chylothorax [1, 17–19]. After the diagnosis of chylothorax has been established, underlying malignancies (in particular lymphoma) must be ruled out by histopathological analysis. Further diagnostic approaches, for example MR-lymphangiography, need to be considered critically in terms of feasibility, patient safety, and clinical benefit. Imaging of the lymphatic system remains challenging. Recently, non-enhanced MR-lymphography has been attempted in nontraumatic chylothorax [20], which, although non-invasive, still requires sedation in young children. It has also been reported that “dual imaging lymphangiography”, combining intermittent digital X-rays and live near-infrared imaging with microsurgical techniques, might be an option for refractory chylothorax [4]. However, as conservative treatment is effective in 80% of pediatric patients, such imaging techniques should be kept for unresponsive or complicated cases [21, 22].

Besides pleural drainage for acute respiratory rescue, long-chain fatty acid-free MCT oral diet, total parenteral nutrition, and the use of somatostatin or its analogue octreotide are common therapeutic approaches [2, 10]. As most reports are based on cases of post-operative chylothorax in children undergoing heart surgery, evidence on the effectiveness of treatment strategies in spontaneous or traumatic chylothorax is still lacking. It seems reasonable to begin therapy with a non-invasive fat-free oral diet and supplementation of medium chain triglycerides in clinically stable patients. Medium-chain triglycerides (saturated fatty acids of eight to twelve carbon chain lengths) are absorbed directly into the portal venous system and bypass the lymphatic drainage, thereby not further enhancing the lymphatic flow [1]. In our patient, we used octreotide early in the treatment algorithm after diet alterations alone did not lower the fluid output (Fig. 2A). In doing so it is important to note that we abstained from beginning a total parenteral nutrition (TPN), which is typically considered the next therapeutic step. The implementation of TPN for toddlers, potentially for periods of several weeks, is particularly arduous and often not feasible in the long term. To the best of our knowledge, this case report is the first to describe foregoing this therapeutic option, in an attempt to avoid potentially stressful effects on young children with spontaneous chylothorax.

Octreotide is a somatostatin analogue which, in comparison to somatostatin, has a longer half-life in circulation and the beneficial option of subcutaneous administration [23]. Both somatostatin and octreotide are routinely used in combination with chest tube drainage and enteric rest in postoperative adult patients with chyle leaks. This has been shown to decrease the need for surgical intervention. In most reports, the benefit of octreotide treatment was seen within 2–3 days [24]. Although the mechanism of action is not completely understood [25], it is known that octreotide reduces intestinal blood flow due to vasoconstriction of the splanchnic circulation, thus reducing gastric, pancreatic, and biliary secretions. Furthermore, fat absorption from the intestine is also reduced [26, 27].It has also been shown that somatostatin receptors SSTR2 and SSTR5 are expressed in the human thoracic duct and that their stimulation may decrease lymphatic flow as well as lymph production [28, 29]. As published recently in a case series, orally administered propranolol, commonly used to treat infantile hemangiomas, seems to be an alternative treatment for chylothorax in children [30]. Benefits of a therapy with propranolol include its easy administration, broad experience, and easy accessibility.

Despite this, if conservative treatments turn out to be ineffective, the invasive management of chylothorax needs to be considered. In the literature pleuroperitoneal shunting, thoracic duct ligation and pleurodesis are described as promising surgical interventions [8, 9, 11, 31].

This case shows that severe episodes of coughing and the resulting elevation of intrathoracic pressure may lead to injury of the thoracic duct and consequently to chylothorax. To the best of our knowledge and based on a detailed review of the available literature, there are no analogous case reports that have been documented to date aligning the association between severe cough and the onset of chylothorax in childhood. In children, several documented cases have reported the occurrence of intrinsic chylothorax subsequent to vomiting, e.g. in two, seven and nine year old children [8, 32, 33] and after trauma [6]. No cases have been reported in children following coughing, although there have been a number of cases elucidating to this rare relationship in the adult population [34, 35]. Considering the history of excessive coughing and immediate onset of dyspnea, the diagnosis of cough-induced chylothorax is in the case of this two year old boy plausible. This case report is however limited by the lack of a prior normal chest X-ray before the incident to rule out a pre-existing condition. Nevertheless, without any pertinent past medical history as well as a recurrence-free interval of more than 2 years after the chylothorax, a predisposing disease is deemed unlikely.

In conclusion, this is a rare case of cough-induced chylothorax in an otherwise healthy toddler. After the failure of first-line conservative treatment, including a strictly fat-free oral diet, we abstained from the generally recommended next therapeutic step of TPN due to the patient’s age. Octreotide proved to be an effective and safe treatment of non-surgical traumatic chylothorax.

## Consent for publication

Informed written consent was obtained from the caregivers of the patient for publication of this case. A copy of the written consent is available for review by the Editor-in-Chief of this journal. The caregivers were involved in the healthcare decisions.

## Competing interests

The authors declare no competing interests.

## Data Availability

All data generated or analyzed in this case report are included in this published article.

## Abbreviations

TPN: Total parenteral nutrition
